# Unraveling the causes of sarcopenia: Roles of neuromuscular junction impairment and mitochondrial dysfunction

**DOI:** 10.14814/phy2.15917

**Published:** 2024-01-15

**Authors:** Yanmei Miao, Leiyu Xie, Jiamei Song, Xing Cai, Jinghe Yang, Xinglong Ma, Shaolin Chen, Peng Xie

**Affiliations:** ^1^ Department of Critical Care Medicine of the Third Affiliated Hospital (The First People's Hospital of Zunyi) Zunyi Medical University Zunyi China; ^2^ Department of Nursing of Affiliated Hospital Zunyi Medical University Zunyi China; ^3^ Department of The First Clinical College Zunyi Medical University Zunyi China

**Keywords:** mitochondrion, neuromuscular junction, Sarcopenia

## Abstract

Sarcopenia is a systemic skeletal muscle disease characterized by a decline in skeletal muscle mass and function. Originally defined as an age‐associated condition, sarcopenia presently also encompasses muscular atrophy due to various pathological factors, such as intensive care unit‐acquired weakness, inactivity, and malnutrition. The exact pathogenesis of sarcopenia is still unknown; herein, we review the pathological roles of the neuromuscular junction and mitochondria in this condition. Sarcopenia is caused by complex and interdependent pathophysiological mechanisms, including aging, neuromuscular junction impairment, mitochondrial dysfunction, insulin resistance, lipotoxicity, endocrine factors, oxidative stress, and inflammation. Among these, neuromuscular junction instability and mitochondrial dysfunction are particularly significant. Dysfunction in neuromuscular junction can lead to muscle weakness or paralysis. Mitochondria, which are plentiful in neurons and muscle fibers, play an important role in neuromuscular junction transmission. Therefore, impairments in both mitochondria and neuromuscular junction may be one of the key pathophysiological mechanisms leading to sarcopenia. Moreover, this article explores the structural and functional alterations in the neuromuscular junction and mitochondria in sarcopenia, suggesting that a deeper understanding of these changes could provide valuable insights for the prevention or treatment of sarcopenia.

## Graphical abstract

We believe that this paper will be of interest to the readership of your journal because the dysfunction of mitochondria and the neuromuscular junction may be one of the key pathophysiological mechanisms leading to sarcopenia. The structural and functional changes of the neuromuscular junction and mitochondria in sarcopenia from the perspective of the neuromuscular junction and mitochondrial pathology might be new targets for the prevention or treatment of sarcopenia in the future.

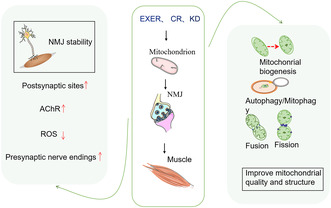

## 1 INTRODUCTION

**FIGURE 1:**
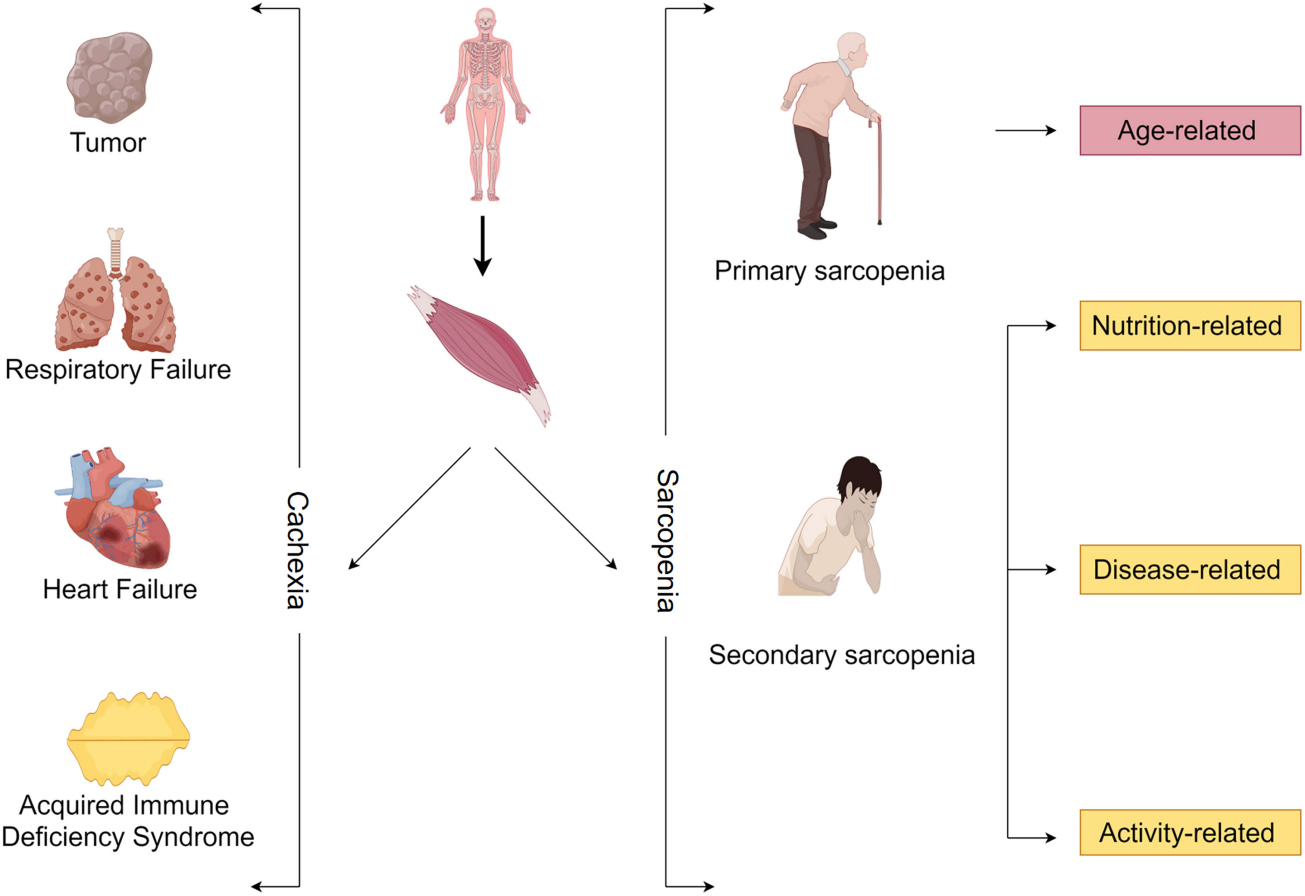
Classification of sarcopenia: primary sarcopenia is mainly age‐related sarcopenia, while secondary sarcopenia includes exercise‐related sarcopenia, disease‐related sarcopenia, and nutrition‐related sarcopenia.

Sarcopenia, a progressive and systemic skeletal muscle disease (Cruz‐Jentoft & Sayer, 2019; Dent et al., 2018), is categorized into primary and secondary types. Primary sarcopenia is age‐associated and has become one of the major health problems in older adults (Gustafsson & Ulfhake, 2021; Papadopoulou, 2020), leading to an increased risk of falls, disability, and mortality (Senior et al., 2015; Westbury et al., 2023). In contrast, secondary sarcopenia is associated with various diseases (Figure 1) (Bauer et al., 2019; Chen et al., 2020; Nishikawa et al., 2016), such as intensive care unit‐acquired weakness (ICU‐AW), amyotrophic lateral sclerosis (ALS), and muscular atrophy due to inadequate exercise and nutrition, and often results in prolonged hospital stays and higher mortality rates (Cacciani et al., 2020; Mitobe et al., 2019; Riancho et al., 2021; Schweickert et al., 2022; Wieske et al., 2015). However, it is important to distinguish between cachexia and sarcopenia. Cachexia, a systemic wasting condition, is typically considered a late‐stage manifestation of chronic diseases, such as cancer, organ failure, or infections (Ferrer et al., 2023). At present, the exact pathogenesis of sarcopenia remains unclear. Nevertheless, some studies have found abnormalities in the morphology of the neuromuscular junction (NMJ) (Carnio et al., 2014; Iyer et al., 2021; Shi et al., 2014; Sirago et al., 2023) and changes in mitochondrial function (Baraldo et al., 2020; Dupuis et al., 2009; Spendiff et al., 2016; Stephenson et al., 2023; Xiao et al., 2020) in individuals with sarcopenia. These findings suggest that impairments in the NMJs and mitochondrial function could be potential contributing factors to the development of sarcopenia (Ahn et al., 2019; Jang et al., 2010).

The NMJ, the contact point between the motor neuron axon and skeletal muscle, is composed of motor nerve endings, perisynaptic Schwann cells, and skeletal muscle fibers (Court et al., 2008; Li et al., 2018; Sanes & Lichtman, 1999). The primary function of this junction is to convert action potentials of presynaptic motor neurons into contraction of postsynaptic muscle fibers. As impairment of the NMJs can lead to muscle weakness or even paralysis (Lepore et al., 2019; Ohkawara et al., 2021; So et al., 2018), NMJ instability is considered to be an initiating and driving factor of the pathology of sarcopenia (Deschenes et al., 2010; Dobrowolny et al., 2021; Rodrigues et al., 2018). In fact, some scholars even describe sarcopenia as a “disorder of the NMJs.” (Alchin, 2014; Gatti et al., 2023) Moreover, mitochondria, which are enriched on both sides of the NMJs (Lee & Peng, 2006; Vos et al., 2010), play an important role in maintaining NMJ stability and transmission (Martinez‐Pena et al., 2021). Mitochondrial dysfunction is increasingly recognized as a critical contributor to NMJ instability, which leads to skeletal muscle atrophy (Anagnostou & Hepple, 2020; O'Connor et al., 2023; Kim et al., 2023; Rygiel et al., 2016). Given that the causal relationship between NMJ instability and mitochondrial dysfunction in sarcopenia remains unclear, this review explores the pathological mechanisms underlying the relationship between the NMJs and mitochondria in sarcopenia. The objective is to provide insights and perspectives that could aid in the prevention or treatment of this condition.

## 2 CHANGES OF THE NMJS AND MITOCHONDRIA IN SARCOPENIA

Recent studies have increasingly demonstrated that NMJ instability is closely related to the occurrence and development of sarcopenia (Ang et al., 2022; Gonzalez‐Freire et al., 2014; Monti et al., 2021). In primary sarcopenia, aging affects the structure and physiological functions of the NMJs, leading to NMJ fragmentation (Kurokawa et al., 1999; Pratt et al., 2021; Valdez et al., 2010), axon remodeling (Smith & Rosenheimer, 1982), reduction of presynaptic vesicles and nerve endings (Rosenheimer, 1990), decreases in both the number (Courtney & Steinbach, 1981) and affinity (Moreira‐Pais et al., 2022; Smith & Chapman, 1987) of acetylcholine receptors (AChRs), and changes in terminal Schwann cells, such as contraction or intrusion into the synaptic cleft (Rahiminezhad et al., 2023; Wang et al., 2007). In secondary sarcopenia, the NMJs also play a key role. NMJ abnormalities, such as axonal depolarization and axonal degeneration, have been observed in patients with ICU‐AW (Batt et al., 2017; Lacomis et al., 1998; Rahiminezhad et al., 2023). Additionally, in patients with chronic progressive external ophthalmoplegia, NMJ abnormalities in the extraocular muscles lead to increased neuromuscular jitter (Zou & Pan, 2022). Moreover, NMJ function is impaired in patients with ALS (Alhindi et al., 2022; Gulino, 2023) and myasthenia gravis (Vecchio et al., 2020).

Mitochondria are highly dynamic organelles with the capacity to modify their function through various mechanisms, including changes in mitochondrial DNA (mtDNA), fusion or fission (Westermann, 2010), and autophagy. In primary sarcopenia, age‐related point mutations in mtDNA are a contributing factor (Wallace, 2013). Mitochondrial fusion proteins 1 and 2 and optic atrophy 1, along with fission proteins such as dynamin‐related protein 1, mitochondrial fission factor, and fission protein 1 (Detmer & Chan, 2007), are critical for maintaining mitochondrial function (Archer et al., 2013). Notably, the inhibition of mitochondrial fusion by knock‐out of mitochondrial fusion proteins 1 and 2 in skeletal muscle can lead to mtDNA deficiency and the accumulation of small muscle sizes (Chen et al., 2010); moreover, mitochondrial autophagy, which facilitates the removal of dysfunctional mitochondria, is essential for the maintenance of muscle mass (Masiero et al., 2009).

Mitochondria have multiple functions, including adenosine triphosphate (ATP) production (Altman et al., 2019), neurotransmitter release (Misgeld & Schwarz, 2017), calcium regulation (Wu, Zheng, et al., 2021), signaling (Ly & Verstreken, 2006), and amino acid and lipid metabolism (Spinelli & Haigis, 2018). Both presynaptic nerve endings and postsynaptic endplate regions are rich in mitochondria (Anagnostou & Hepple, 2020; Lepore et al., 2019; Spendiff et al., 2016), which provide a large amount of ATP for neuromuscular transmission. Moreover, regulation of the mitochondrial network plays a key role in determining muscle size (Herbst et al., 2007; Rahman & Quadrilatero, 2021; Romanello & Sandri, 2021; Xu et al., 2022). In critically ill patients with ICU‐AW, there is evidence of ultrastructural damage and functional impairment of mitochondria in skeletal muscles (Batt et al., 2017; Felix Klawitter, 2023; Friedrich et al., 2015). Additionally, an animal study also found that mitochondrial swelling, mitochondrial cristae remodeling, and membrane rupture in motor neuron terminals were followed by functional failure of the NMJs in aging rats (García et al., 2013). These structural changes in skeletal muscle mitochondria both in critically ill patients and aging rats may affect the function of the NMJs (Brischigliaro et al., 2023). However, other researchers have demonstrated that there is no direct relationship between mitochondrial morphology and NMJ function. For example, one *Drosophila melanogaster* study has shown that mitochondrial fragments with basic normal biological capacity could be produced in the nervous system, and the flies could survive in the presence of a small number of mitochondria; (Trevisan et al., 2018) this finding suggests that these mitochondrial fragments are functional, and a small number of mitochondria with bioenergetic activity can maintain normal neural function. Taken together, the aforementioned evidence suggests a close relationship between mitochondria and the NMJs (Lee & Peng, 2008; Rikhy et al., 2007; Wilson et al., 2018). However, whether they unilaterally or collectively contribute to sarcopenia remains unknown.

**FIGURE 2:**
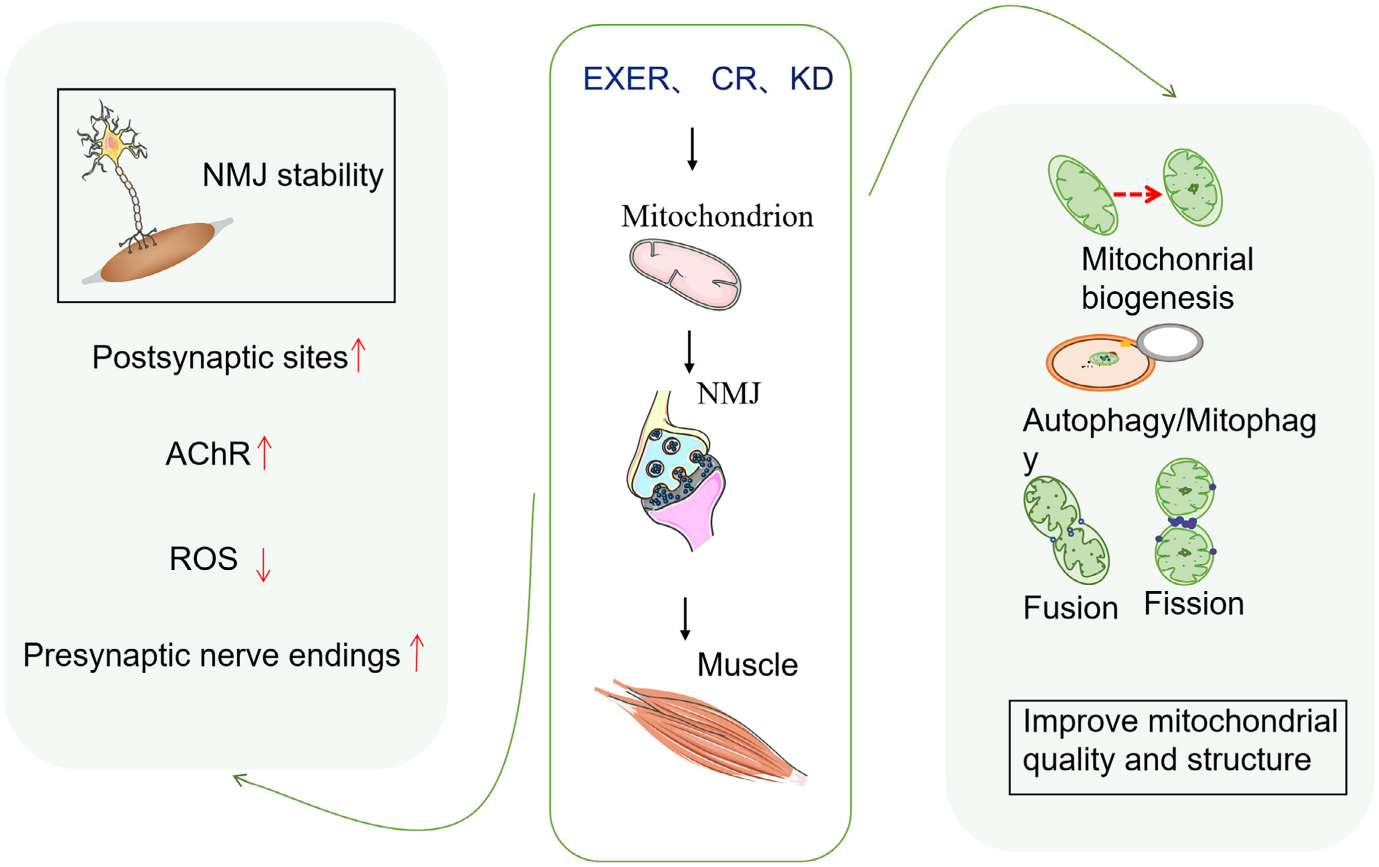
Exercise, a ketogenic diet, and a calorie‐restricted diet may prevent sarcopenia to some extent. CR, caloric restriction; EXER, exercise; KD, ketogenic diet.

Despite numerous studies highlighting the significant roles of the NMJs and mitochondria in sarcopenia, only two interventions have been currently reported to effectively manage NMJs and mitochondrial disruption in sarcopenia: exercise and calorie restriction (CR) (Jang et al., 2012; Shen et al., 2023). Exercise improves mitochondrial function and NMJ health (Kim et al., 2013; Lee et al., 2019). CR reduces proton leakage and reactive oxygen species (ROS) production in skeletal muscle mitochondria. In addition, CR enhances the expression of genes involved in ROS scavenging and alters the fatty acid composition of mitochondrial membranes, thereby reducing lipid oxidation and proton leakage (Drew et al., 2003; Short et al., 2005). Moreover, CR can directly affect NMJs; for example, it increases the number of sarcoplasmic ducts, leading to a greater number of postsynaptic sites that exhibit young structures (Jang et al., 2012). Another promising strategy involves the administration of ketone bodies or the activation of ketogenesis (Goossens et al., 2019; Yakupova et al., 2022). This approach is gaining interest because a ketogenic diet or in vitro ketogenic supplementation with ketone bodies can increase mitochondrial activity in young animals and improve mitochondrial mass and function in older animals (Cioni et al., 2019). However, the potential positive effect of a ketogenic diet on sarcopenia remains to be explored (Figure 2).

## 3 MITOCHONDRIAL DYSFUNCTION IS NOT DIRECTLY RELATED TO NMJ IMPAIRMENT

Although mitochondria are generally believed to be closely related to the function of the NMJs, some studies have presented opposite conclusions. For example, the improvement of redox‐dependent sarcopenia by mitochondrial peroxiredoxin‐3 overexpression did not correspondingly alleviate NMJ damage (Ahn et al., 2022), possibly because other sources of ROS (i.e., nicotinamide adenine dinucleotide phosphate oxidase or cytosolic phospholipase A2) located in the sarcolemma and/or associated with binding lipid hydroperoxides were involved in NMJ damage (Pharaoh et al., 2020). Additionally, another study suggests that mitochondrial dysfunction and oxidative stress might directly affect myosin interaction and force generation but do not significantly affect the stability of the NMJs (Carnio et al., 2014). This finding indicates no direct relationship between mitochondrial dysfunction and NMJ instability.

## 4 MITOCHONDRIAL DYSFUNCTION PRECEDES CHANGES IN THE NMJS

### 4.1 Role of mitochondria in the NMJs

Mitochondria are closely related to NMJ function (Lee & Peng, 2008; Rikhy et al., 2007; Wilson et al., 2018). While only approximately 50% of active synapses in the central nervous system of adult rodents contain mitochondria, NMJs are notably enriched in tightly packed mitochondrial networks (Altman et al., 2019; Misgeld & Schwarz, 2017). Mitochondria are present both pre‐ and post‐synaptically at NMJs (Osborne, 1967). Following pathological changes in the mitochondria, the production of ATP and acetylcholine and the regulation of calcium and ROS are disturbed, thus affecting NMJ transmission (Batt et al., 2017; Wu, Zhao, et al., 2021), These findings indicate that mitochondria play a unique role in maintaining NMJ function and stability.

#### 4.1.1 Effect of mitochondrial changes on the NMJs

Mitochondria affect the NMJs through a variety of pathways (Table 1). Patients with sarcopenia resulting from mitochondrial myopathy have decreased synaptic vesicles at the presynaptic side, abnormal mitochondria aggregation at both the presynaptic and postsynaptic sides, and decreased NMJs (Lu et al., 2019), indicating the presence of pathological changes at the NMJs in sarcopenia. For example, multiple or single deletions of mtDNA can lead to alterations in myelin sheaths and crystal‐like inclusions in Schwann cells at NMJs (Pareyson et al., 2013). However, it is still controversial whether pathological changes of the NMJs precede or follow muscular changes (Lepore et al., 2019). Animal studies have suggested that mitochondrial alterations might precede pathological changes in muscles and NMJs in neuromuscular diseases. For example, a study of transgenic mice overexpressing fused in sarcoma (FUS), a marker of pathologies in FUS‐related ALS and frontotemporal dementia, showed that mitochondrial defects preceded NMJ instability (So et al., 2018). Similarly, mice with the p.Ser59Leu variant in the mitochondrial protein CHCHD10, which were used to model mitochondrial myopathy with unstable mtDNA, showed mitochondrial changes in skeletal muscles before NMJ instability, motor neuron degeneration, and high fragmentation of the motor endplate (Genin et al., 2019). This finding supports the view that the pathological changes of mitochondria precede NMJ alterations, affecting both the structure and function of the NMJs (Dobrowolny et al., 2018; Pollari et al., 2014).

**TABLE 1:** Effects of mitochondrial changes on neuromuscular function.

Types of sarcopenia	Mitochondria	NMJ	Document
Amyotrophic Lateral Sclerosis, frontotemporal dementia	Mitochondrial destruction	A loss of synaptic vesicles and synaptophysin protein	So et al. (2018)
Motor Neuron Disease	Structural instability of inner mitochondrial membrane and OXPHOS deficiency	NMJ highly fragmented	Genin et al. (2019)
Amyotrophic Lateral Sclerosis	The local synthesis of nuclear encoded mitochondrial protein is inhibited	NMJ disruption	Altman et al. (2021)
Amyotrophic Lateral Sclerosis	Mitochondrial uncoupling exacerbated	NMJ regeneration and functional recovery were profoundly delayed	Dupuis et al. (2009)
Parkinson's Disease	Increased mitochondrial fragmentation	Abnormal behavior and structural changes of NMJ	Zhu et al. (2013)
	Mitochondrial protein S‐nitrosation	Protects against ischemia reperfusion‐induced denervation at NMJ	Wilson et al. (2018)
Primary Myopenia	The number and speed of mitochondrial NADH decrease increase	NMJ deterioration or loss of mass	Su et al. (2021)
Myasthenia Gravis, Amyotrophic Lateral Sclerosis	Mitochondrial CHCHD10 protein mutation	Motion defect, abnormal neuromuscular conduction and NMJ structural damage	Xiao et al. (2020)
Congenital Myasthenic Syndrome	Mutation of mitochondrial citrate carrier SLC25A1	Impaired Neuromuscular Transmission	Chaouch et al. (2014)
	Mitochondrial drp1 mutation	Mobilization damage of the reserve pool container at the NMJ	Verstreken et al. (2005)
Chronic Intermittent Hypoxia	Mitochondrial swelling and SOCS3 upregulation	NMJ deterioration	Bannow et al. (2022)

Along with affecting energy supply, mitochondrial dysfunction increases the production of free radicals and ROS, triggering a vicious cycle of damage to macromolecules and organelles (Alizadeh Pahlavani et al., 2022; Martin et al., 2021). Mitochondrial dysfunction and increased ROS production in aged muscle have been identified as two key factors contributing to NMJ deterioration and primary sarcopenia. A study directly examining the effect of chronic oxidative stress on skeletal muscle in vivo found that aged mice lacking the antioxidant enzyme copper‐zinc superoxide dismutase exhibited a significant increase in mitochondrial numbers near the NMJs. However, mitochondrial function was significantly impaired, with broken AChRs and impaired NMJ function. Interestingly, the study also found that mitochondrial function within the muscle decreased during aging, as well as in response to altered neuronal redox status prior to NMJ deterioration or loss of muscle mass and force (Zorov et al., 2000). These findings suggest that mitochondrial defects might play a role in the development of sarcopenia, independent of denervation. Moreover, in patients with ICU‐AW, inflammation, hyperglycemia, and free radicals exacerbate mitochondrial damage, leading to impaired oxygen utilization in mitochondria and thereby affecting NMJ function (Friedrich et al., 2015). Additionally, scavenging peroxides produced by mitochondria can effectively prevent mitochondrial dysfunction and subsequent NMJ destruction associated with muscle atrophy due to denervation. This finding supports mitochondrial hydrogen peroxide as an important effector in NMJ changes associated with sarcopenia (Xu et al., 2021). These previous studies collectively indicate that mitochondria play a crucial role in maintaining the structural and functional integrity of the NMJs, and pathological changes in mitochondrial function or structure are directly linked to the corresponding functional damage of the NMJs.

## 5 NMJ ABNORMALITIES AFFECT MITOCHONDRIAL FUNCTION

The normal structure and function of the NMJs play important roles in mitochondrial function (Table 2) (Sirago et al., 2023). Mitochondria are transported from the main biosynthetic sites in the cell body along the cytoskeleton, traveling down the axon to the synaptic terminal (Barnhart, 2016; González & Couve, 2014). Changes in the structure of the NMJs can affect the location and number of mitochondria reaching the synapses, thereby affecting NMJ function and aggravating sarcopenia. In healthy adult animals, local protein synthesis occurs in the axons and synapses of NMJs (Hafner et al., 2019), playing an important role in the structural and functional integrity of mitochondria. However, it is unclear how the local protein synthesis in axons maintains the mitochondrial structure and function. Moreover, nuclear‐encoded mitochondrial messenger RNAs (mRNAs) have been identified in the axon at the NMJs (Maciel et al., 2018), and they are translated near the mitochondria within the axon (Cioni et al., 2019). Notably, these nuclear‐encoded mitochondrial genes are the most abundant mRNAs in the axonal transcriptome of human motor neuron (Maciel et al., 2018). However, the exact mechanism by which these nuclear‐encoded mitochondrial mRNAs are transported to the synapses, as well as the process of their translation at these sites, need to be further investigated.

**TABLE 2:** Effect of NMJ on mitochondria.

NMJ	Mitochondria	Document
Changes of axonal endoplasmic reticulum in NMJ	Affect the local supply of mitochondria	González and Couve (2014); Barnhart (2016)
Axis protein synthesis disorder	Promoting mitochondrial damage	Hafner et al. (2019)
Axon‐enriched nuclei in NMJ encode mitochondrial mRNA	Maintain mitochondrial structure and function	Maciel et al. (2018); Cioni et al. (2019)

A study showed that accumulation of mislocalized TAR‐DNA‐binding protein (TDP)‐43 at the NMJs, a phenomenon observed in the motor neurons of approximately 95% of ALS patients, impaired local translation of nuclear‐encoded mitochondrial proteins and inhibited local protein synthesis in distal axons and NMJs, thereby leading to decreases in nuclear‐encoded mitochondrial protein levels in axons and synapses. Interestingly, the clearance of axonal TDP‐43 increases the level of nuclear‐encoded mitochondrial proteins in axons and synapses (Altman et al., 2021). These findings indicate that the normal structure and function of the NMJs are partly responsible for maintaining healthy mitochondria, while dysfunction of the NMJs can adversely affect structural and functional integrity of mitochondria in sarcopenia.

## 6 CONCLUSIONS

**FIGURE 3:**
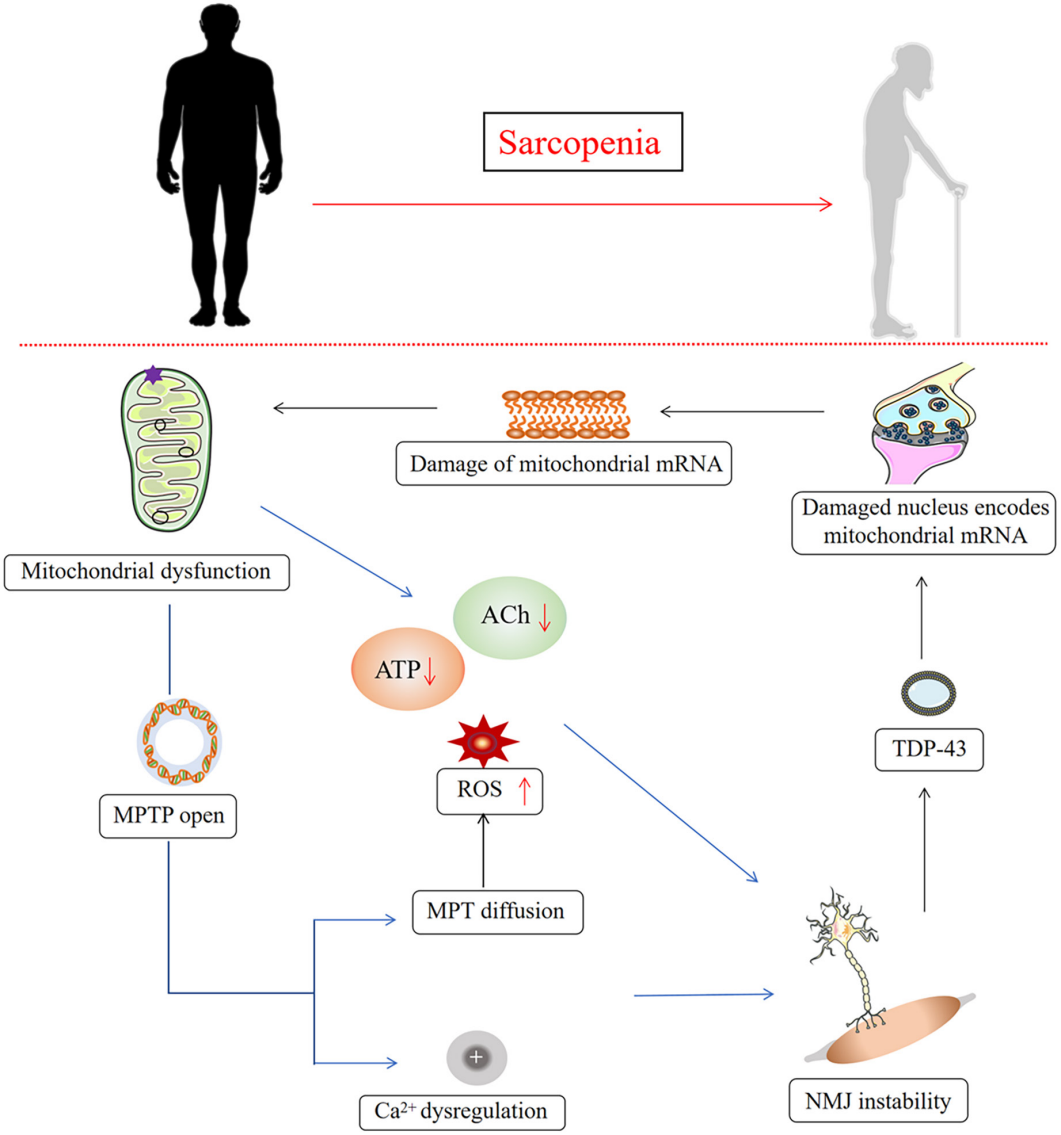
Sarcopenia. The interaction between mitochondria and the neuromuscular junction may be an important pathogenesis of sarcopenia. ACh, ROS, acetylcholine; reactive oxygen species; ATP, adenosine 5′‐triphosphate; MPTP, mitochondrial permeability transition pore; MPT, mitochondrial phosphate transporter; NMJs, neuromuscular junction.

Mitochondria and the NMJs have a close and complex relationship, with damage to one often impacting the other. Most current evidence supports the idea that mitochondrial dysfunction usually precedes NMJ instability in both age‐related sarcopenia and disease‐associated sarcopenia. However, few studies have suggested that NMJ impairment might affect mitochondrial function or that their relationship is not directly causal. The reasons behind the discrepancy are still unclear and warrant further investigation (Figure 3). At present, regular exercise (Carol & Hodgson, 2022; Collao et al., 2023; Jacob et al., 2023) a ketogenic diet (Goossens et al., 2019; Yakupova et al., 2022), and a calorie‐restricted diet (Faulks et al., 2006; Jang et al., 2012) have shown promise in preventing or mitigating NMJs and mitochondrial damage in sarcopenia (Deschenes et al., 2022; Lee et al., 2019; Stockinger et al., 2017). In conclusion, understanding the interplay between mitochondria and NMJs could provide valuable insights into the development of treatments or preventive strategies for sarcopenia.

## AUTHOR CONTRIBUTIONS

YM, LX, JS, XM, SS, and PX performed the literature search, wrote the first draft of the manuscript, and which was critically reviewed by PX. All authors contributed to the article and approved the submitted version.

## FUNDING INFORMATION

This work was supported by the National Natural Science Foundation (Grant Nos. 82060359 and 82360382) of China; Guizhou Province Social Development Project: Qiankehe [2021] General 088; Key Project of Guizhou Natural Science Foundation: Qiankehe Fundamentals ZK [2022] Key 049; Guizhou Province Excellent Youth Science and Technology Talent Project: Qiankehe Platform Talent [2021] No. 5648; Zunyi Excellent Youth Science and Technology Talent Project: Zunyou Qingke (2020) No. 2; Zunshi Kehe H8 Zi (2020) No. 144; Yuan Ke Zi (2020) No. 13; Zunyi Medical University College Student Innovation and Entrepreneurship Training Program (No. ZYDC202202048).

## CONFLICT OF INTEREST STATEMENT

The authors declare that the research was conducted in the absence of any commercial or financial relationships that could be construed as a potential conflict of interest.

## ETHICS STATEMENT

Not applicable.
